# Editorial: Innovative strategies for overcoming resistance in tumor angiogenesis therapies

**DOI:** 10.3389/fphar.2026.1801844

**Published:** 2026-02-23

**Authors:** Peace Mabeta, Vanessa Steenkamp, José Díaz-Chávez

**Affiliations:** 1 Department of Physiology, University of Pretoria, Pretoria, South Africa; 2 Department of Pharmacology, University of Pretoria, Pretoria, South Africa; 3 Instituto Nacional de Cancerologia, Mexico City, Mexico

**Keywords:** anti-angiogenic targeted therapy, biomarkers, cancer, drug resistance, tumor angiogenesis

The advent of anti-angiogenic therapies has significantly impacted oncological care, providing enhanced prospects for disease management and patient survival across various solid tumour types. However, the clinical efficacy of these agents is frequently undermined by the emergence of resistance, which remains a formidable obstacle to durable therapeutic success. As tumour cells adapt to angiogenic blockade, they exploit alternative mechanisms to sustain their blood supply, evade immune surveillance, and metastasise (Mabeta and Pepper, 2017; Kuo et al., 2024; Wang et al., 2025). The need to surmount these adaptive responses has fuelled a wave of innovative research, the highlights of which are showcased in this Research Topic section.

This editorial synthesises the findings and significance of seven key articles, each offering fresh insights into the mechanisms underpinning resistance to angiogenesis therapies and proposing novel strategies to overcome them. By integrating molecular, clinical, and translational perspectives, these contributions collectively point the way towards more effective, personalised, and durable anti-angiogenic regimens for cancer patients.

In a study of the tumour-suppressive role of KCTD10 in non-small cell lung cancer (NSCLC), the authors demonstrate that KCTD10 inhibits metastasis and angiogenesis by promoting the ubiquitination and proteasomal degradation of β-catenin, a central mediator of the Wnt signalling pathway. Their findings highlight the potential of targeting the KCTD10–β-catenin axis to overcome resistance to conventional anti-angiogenic therapies, which often fail due to compensatory Wnt/β-catenin signalling.

On the other hand, two comprehensive systematic reviews and meta-analyses evaluate the efficacy and safety of incorporating bevacizumab into neoadjuvant chemotherapy regimens for ovarian cancer. The pooled data suggest that bevacizumab enhances pathological response rates and may confer progression-free survival benefits, albeit with an increased risk of adverse events such as hypertension and proteinuria. These findings underscore the need for careful patient selection and monitoring, as well as the potential value of biomarkers to predict response and toxicity. Importantly, the analyses highlight the persistent challenge of resistance, as not all patients derive sustained benefit from bevacizumab-based combinations.

Another exciting focus in this topic is explored in a retrospective clinical study that addresses an underexplored patient population: the elderly with NSCLC, where the combination of a recombinant human vascular endothelial growth factor inhibitor with an anti-PD-1 immune checkpoint inhibitor was assessed for efficacy and safety in patients aged 80 years and older. The results indicate that the combination is both feasible and tolerable, with evidence of anti-tumour activity and manageable toxicity profiles. The study provides early support for integrating anti-angiogenic and immunotherapeutic strategies, particularly in populations often excluded from clinical trials. Moreover, the addition of bevacizumab to gefitinib and chemotherapy was also explored as a first-line regimen for patients with EGFR L858R-mutant advanced NSCLC in another study. The triplet therapy demonstrated promising efficacy, with improved progression-free survival compared to historical controls, and a safety profile consistent with known toxicities. The findings support the concept that simultaneous targeting of angiogenic, and proliferative pathways may delay the onset of resistance and prolong clinical benefit in molecularly defined subsets of lung cancer.

Another systematic review synthesises the evidence for vasculogenic mimicry (VM) as a mechanism of resistance to anti-angiogenic therapies in NSCLC. This review identifies key molecular drivers of VM, including hypoxia-inducible factors and matrix metalloproteinases, and discusses emerging therapeutic strategies to disrupt VM formation. Targeting VM may represent a critical adjunct to conventional anti-VEGF approaches, particularly in resistant or relapsed disease.

Finally, a meta-analysis and trial sequential analysis evaluate the impact of combining anti-angiogenic agents with chemotherapy in both platinum-sensitive and platinum-resistant ovarian cancer. The results indicate that combination therapy yields superior response rates and progression-free survival compared to chemotherapy alone, with an acceptable safety profile. However, the incremental benefits are modest, and resistance remains a significant barrier, particularly in the platinum-resistant cohort. The authors call for the development of predictive biomarkers and novel drug combinations to further enhance outcomes.

In conclusion, the contributions to this Research Topic section underscore the dynamic and multifaceted nature of resistance to tumour angiogenesis therapies. By identifying new molecular targets, validating innovative combination therapies, and emphasising the significance of patient stratification, these studies provide guidance for developing more effective and sustainable anti-angiogenic strategies. Overcoming resistance will require continued collaboration across disciplines, integration of emerging scientific insights, and a steadfast commitment to patient-centred care.
